# Risk Factors Associated with HIV Acquisition in Males Participating in HIV Vaccine Efficacy Trials in South Africa

**DOI:** 10.1007/s10461-023-04025-z

**Published:** 2023-03-16

**Authors:** Mookho Malahleha, Fatima Laher, Athmanundh Dilraj, Philip Smith, Glenda E. Gray, Doug Grove, Jackline A. Odhiambo, Michele P. Andrasik, Nicole A. Grunenberg, Zoe Moodie, Yunda Huang, Bhavesh R. Borate, Kevin M. Gillespie, Mary Allen, Millicent Atujuna, Nishanta Singh, Dishiki Kalonji, Graeme Meintjes, Phillip Kotze, Linda-Gail Bekker, Holly Janes

**Affiliations:** 1 Setshaba Research Centre, Soshanguve, Tshwane, South Africa; 2 Synergy Biomed Research Institute, East London, Eastern Cape South Africa; 3 Perinatal HIV Research Unit, Faculty of Health Sciences, University of the Witwatersrand, Johannesburg, South Africa; 4 The Desmond Tutu HIV Centre, Institute for Infectious Disease and Molecular Medicine, Faculty of Health Science, University of Cape Town, Cape Town, South Africa; 5 South African Medical Research Council, Cape Town, South Africa; 6 Vaccine and Infectious Disease Division, Fred Hutchinson Cancer Research Center, Seattle, WA USA; 7 Vaccine Research Program, Division of AIDS, National Institute of Allergy and Infectious Diseases, National Institutes of Health, Bethesda, MD USA; 8 HIV and other Infectious Diseases Research Unit, South African Medical Research Council, Durban, South Africa; 9 Wellcome Centre for Infectious Diseases Research in Africa, Cape Town, South Africa; 10 Qhakaza Mbokodo Research Clinic, Ladysmith, South Africa; 11 Setshaba Research Centre, 2088 Block H, Soshanguve, Pretoria, 0152 South Africa

**Keywords:** HIV incidence, HIV risk factors, Sexual behavior, Males at birth, Non-heterosexual identity

## Abstract

In South Africa, HIV acquisition risk has been studied less in people assigned male at birth. We studied the associations between risk behaviors, clinical features and HIV incidence amongst males in two South African HIV preventive vaccine efficacy trials. We used Cox proportional hazards models to test for associations between demographics, sexual behaviors, clinical variables and HIV acquisition among males followed in the HVTN 503 (n = 219) and HVTN 702 (n = 1611) trials. Most males reported no male sexual partners (99.09% in HVTN 503) or identified as heterosexual (88.08% in HVTN 702). Annual HIV incidence was 1.39% in HVTN 503 (95% CI 0.76–2.32%) and 1.33% in HVTN 702 (95% CI 0.80–2.07%). Increased HIV acquisition was significantly associated with anal sex (HR 6.32, 95% CI 3.44–11.62), transactional sex (HR 3.42, 95% CI 1.80–6.50), and non-heterosexual identity (HR 16.23, 95%CI 8.13–32.41) in univariate analyses and non-heterosexual identity (HR 14.99, 95% CI 4.99–45.04; p < 0.01) in multivariate analysis. It is appropriate that prevention efforts in South Africa, although focused on the severe epidemic in young women, also encompass key male populations, including men who have sex with men, but also men who engage in anal or transactional sex.

**Supplementary Information:**

The online version contains supplementary material available at 10.1007/s10461-023-04025-z.

## Introduction

Most of the 84.2 million people who have acquired HIV, and the 40.1 million people who have perished from AIDS, live in sub-Saharan Africa [1]. In South Africa, which bears the largest HIV burden worldwide, the epidemic is mainly spread heterosexually, and young women experience disproportionate acquisition [2]. As a result, HIV research and interventions have focused on women in this region. Increasingly though, it is being recognized that addressing risk behaviors of men could contribute to curbing HIV transmission [3].

In South Africa, about 2.4 million men aged 15 years and older were living with HIV in 2021 [4]. HIV epidemiology in men is different compared to women in South Africa: incidence in men peaks at a later age [5], prevalence is higher in older compared to younger individuals [6] and clinical disease outcomes are worse due to low or delayed access to health care and reduced HIV testing appetite [7].

In addition to young women, recognized key populations for HIV prevention efforts in South Africa include sex workers and men who have sex with men (MSM). There has also been a description of a key population of high-risk heterosexual men, whose risk factors include condomless sex with multiple female partners, and whose alcohol use has been described as associated with HIV acquisition [8].

In South Africa, high HIV prevalence has been observed in older heterosexual men with age-disparate sexual relationships [6, 9], in younger men with multiple female sex partners [6], and in MSM [10, 11]. There are limited health data for MSM and transgender women (TGW) populations in sub-Saharan Africa. Although the HIV epidemic is largely driven by heterosexual transmission in South Africa, a disproportionate burden remains with the MSM population whose prevalence is estimated to be between 25 and 49% [10, 11].

HIV vaccine trials extensively document risk factors and perform periodic HIV testing, providing useful and detailed longitudinal data to study factors associated with HIV acquisition. In this paper, we maximized data by pooling the South African HIV vaccine efficacy trials which enrolled individuals assigned male sex at birth. Both trials enrolled participants at risk of HIV. Although the trials were conducted at different points in time and measured some of the risk factors somewhat differently, they provide an unprecedented opportunity to study factors associated with HIV acquisition in the understudied population of South African men. We describe the association between HIV acquisition with the demographic, behavioral and clinical factors of individuals assigned male sex at birth (heterosexual men, MSM and TGW) enrolled in HVTN 503 and HVTN 702.

## Methods

### Study Design

In our retrospective analysis, we pooled data from participants born male (hereafter, ‘males’) enrolled in HIV vaccine efficacy trials in South Africa. Only two HIV vaccine efficacy trials in South Africa included males, so both were included in the analysis: HVTN 503/503S (NCT00413725) and HVTN 702 (NCT02968849) (see CONSORT Diagram in Supplementary Materials: Fig. S1) [12, 13]. Both trials were randomized double-blinded placebo-controlled clinical trials which enrolled healthy 18–35-year-old males and females at risk of HIV acquisition. Both trials and this analysis were approved by the research ethics committees of University of the Witwatersrand (60,504; 160208B), University of Cape Town (299/2013; 218/2006; 622/2016; 623/2016), South African Medical Research Council (EC020-7/2016), University of KwaZulu-Natal (T088/06; T090/06; BFC479/16), University of Limpopo (MREC/P/129/2008:CR) and Sefako Makgatho University (SMUREC/P/192/2016:CR).

### Participants and Data Collection

Participants were enrolled into HVTN 503, a phase 2b trial, between January to October 2007. HVTN 503 enrollment was stopped early and the 801 participants were unblinded upon evidence of vaccine futility in another trial of the same investigational vaccine, MRK-Ad5. The HVTN 503 trial design and primary results are published [12]. In 2013, a 2-week substudy called HVTN 503S was conducted to assess HIV acquisition and behavioral risk factors amongst former HVTN 503 participants; these study details and results are also published [14]. Because analyses of HVTN 503/503S suggested a potential vaccine-induced increase in HIV risk in males, our analysis includes data from HVTN 503 participants randomized only to placebo. We included 219 placebo-recipient males in the modified intention to treat (MITT) cohort, 14 of whom acquired HIV during follow-up (HVTN 503 and HVTN 503S, hereafter ‘HVTN 503’), with a total of 6 years median follow-up.

The HVTN 702 phase 2b/3 trial enrolled 5407 participants from October 2016 to June 2019. In 2020, an interim analysis concluded that the ALVAC/gp120/MF59 vaccine regimen was ineffective at preventing HIV acquisition. Vaccinations were stopped; participants were unblinded and followed up for a year. HVTN 702 methods and primary results have been published [15]. Given the absence of an apparent effect of the vaccine on clinical endpoints, this analysis includes both vaccine and placebo recipients from HVTN 702. We included 1611 males from the MITT cohort, 37 of whom acquired HIV during the trial, with a median follow-up of 1.78 years.

At screening, participants in both trials completed a medical history, physical examination, behavioral risk assessment questionnaire, demographics questionnaire, and circumcision status assessment.

### Measures

We collated baseline data regarding demographics (age, race, region, marital/main partner status), sexual behavior [(sexual orientation, reported male partners, anal sex (with female or male partners), exchange of sex for money/gifts, sex with a partner living with HIV)] and clinical characteristics [(genital discharge, genital sores, sexually transmitted infections (STIs), body mass index, and circumcision at baseline)].

Between the two trials, there were some differences in the data collection methods. The timeframe reference for sexual behavior recall was the previous 6 months in HVTN 503, but it was the previous 30 days in HVTN 702. Gender identity and sexual orientation were not collected in HVTN 503, but were collected in HVTN 702. Baseline circumcision status in HVTN 503 was assessed by physical examination or self-report, and then corrected if status via physical examination in HVTN 503 was different, as previously described [14]; baseline circumcision status was assessed by physical examination or self-report in HVTN 702. In HVTN 503, but not HVTN 702, participants were tested for HSV2 antibodies at baseline via Western Blot. At enrollment in HVTN 702, but not in HVTN 503, polymerase chain reaction (PCR) for *Chlamydia trachomatis* and *Neisseria gonorrhoeae* was conducted on urine samples and rectal swabs for MSM. In HVTN 702, but not in HVTN 503, syphilis serology (rapid plasma reagin with reflex Treponema pallidum hemagglutinin) was done at enrollment. In HVTN 503, HIV testing was done at months 0, 3, 8, 13, 16, 19, 22, 26, 29, 33, 36, 39, 43 and 46, and HIV was diagnosed according to the HVTN HIV-1 diagnostic algorithm including HIV-1 Western Blot (WB) and HIV-1 RNA PCR if diagnostic enzyme immunoassay (EIA) was positive. In HVTN 702, HIV testing was done at months 0, 3, 6, 9, 12, 15, 18, 21, 24, 27, 30, 33 and 36, using an updated HVTN diagnostic algorithm of a fourth-generation HIV 1/2 EIA or chemiluminescent microparticle immunoassay with confirmatory PCR.

### Statistical Methods

Distributions of categorical baseline variables were summarized using frequencies, and medians and interquartile ranges were used for continuous variables. Cumulative incidence of HIV infection was estimated using the Kaplan–Meier method. Cox proportional hazards regression was used to associate baseline variables with HIV outcomes, based on data pooled across the two studies and with different baseline hazards for each study and randomization group (HVTN 503 placebo vs. HVTN 702 placebo vs. HVTN 702 vaccine). Associations were measured using hazard ratios (HRs). Baseline variables were evaluated univariately, and a pre-specified multivariate model was fitted using eight variables found to predict HIV risk in at least three previously published studies of men in Africa (see Supplementary Materials Sect. 2). Wald tests were used for inference. The Holm method was used to account for multiplicity, controlling the overall family-wise error rate at 0.05 across the variables assessed univariately. Multiple imputation was used to fill in missing baseline covariate data. Details of the imputation procedure are in the Supplementary Materials Sect. 3. Results were combined across imputed datasets using Rubin’s rules [16].

The Super Learner method was used to develop an HIV risk score, and performance of the model was evaluated using the area under the ROC curve (AUC) (details in Supplementary Materials).

## Results

### HIV Risk Factors and Incidence

In total, data from 1830 males were eligible for this analysis: 219 from HVTN 503 and 1611 from HVTN 702 (Table 1). In HVTN 702, which assessed gender identity, most males at birth identified as male at enrollment (99%); among the remainder, 6 identified as female (0.37%), 2 as gender-variant (0.12%), 10 as transgender female (0.62%), and 2 preferred not to answer (0.12%). Male participants were generally young [median age 22 years in HVTN 503 (IQR 20–26) and 26 years in HVTN 702 (IQR 22–30)] and most had low or normal body mass index at enrollment (91% for HVTN 503, 84% for HVTN 702). Most were Black (98% in HVTN 503, 99% in HVTN 702). While in HVTN 503 the majority of male participants were recruited from central South Africa (79%; Rustenburg, Klerksdorp, Medunsa, Soshanguve, Soweto-Bara, Soweto-Kliptown, Tembisa sites), in HVTN 702 male participants were recruited more broadly, with 52% from central South Africa, 32% from KwaZulu-Natal (Durban-eThekwini, Durban-Isipingo, Durban-Verulam, Ladysmith), and 16% from the Western or Eastern Cape (Cape Town-Emavundleni, Cape Town-Khayelitsha, Mthatha). STIs were prevalent at baseline, with 15% of males in HVTN 503 testing positive for HSV2 and 14% of males in HVTN 702 testing positive for syphilis, *Neisseria gonorrhoeae*, or *Chlamydia trachomatis*. The most common self-reported behavioral risk factors were not being married or not having a main sexual partner (32% in HVTN 503 and 11% in HVTN 702), not living with a spouse or main partner (34% in HVTN 503 and 68% in HVTN 702), having two or more sexual partners (52% in HVTN 503 and 69% in HVTN 702), sex with an HIV-positive partner in HVTN 702 (54%) although this was very uncommonly reported in HVTN 503 (1.4%), and sex with alcohol/drug use (37% in HVTN 503 and 59% in HVTN 702). 30% of HVTN 503 males were circumcised at baseline compared to a much higher proportion of males in HVTN 702 who were circumcised at baseline (53%) (Table 2); this trial occurred after a rollout of voluntary medical male circumcision in South Africa in the 2010s.

**Table 1:** Distribution of baseline demographic variables by study

Baseline variable	HVTN 503/503S (N = 219)	HVTN 702 (N = 1611)
Age, years		
18–21	98 (44.75%)	320 (19.86%)
22–25	66 (30.14%)	435 (27.00%)
26–35	55 (25.11%)	856 (53.13%)
Median (25%ile, 75%ile)	22 (20, 26)	26 (22, 30)
Race		
Black	215 (98.17%)	1587 (98.51%)
Other	3 (1.37%)	0 (0.00%)
White	1 (0.46%)	1 (0.06%)
Asian	0 (0.00%)	1 (0.06%)
Colored/Mixed	0 (0.00%)	18 (1.12%)
Multiple reported	0 (0.00%)	4 (0.25%)
Body mass index (BMI)		
Less than 18.5	46 (21.00%)	232 (14.40%)
18.5 to less than 25	153 (69.86%)	1121 (69.58%)
25 to less than 30	15 (6.85%)	193 (11.98%)
30 or higher	5 (2.28%)	65 (4.03%)
Study site geographic region^a^		
Central	173 (79.00%)	831 (51.58%)
KwaZulu-Natal	15 (6.85%)	522 (32.40%)
West/East Cape	31 (14.16%)	258 (16.01%)

Number (percent) in each category

^a^Kwa-Zulu Natal includes sites Durban-eThekwini, Durban-Isipingo, Durban-Verulam, Ladysmith; Central South Africa includes sites Rustenburg, Klerksdorp, Medunsa, Soshanguve, Soweto-Bara, Soweto-Kliptown, Tembisa; Western/Eastern Cape includes sites Cape Town-Emavundleni, Cape Town-Khayelitsha, Mthatha

**Table 2:** Distribution of baseline behavioral and clinical variables by study

Baseline variable	HVTN 503/503S (N = 219)	HVTN 702 (N = 1611)
Heterosexual sexual orientation		
No	0 (0.00%)	192 (11.92%)
Yes	0 (0.00%)	1419 (88.08%)
Missing	219 (100.00%)	0 (0.00%)
Male sexual partner(s)		
No	217 (99.09%)	0 (0.00%)
Yes	2 (0.91%)	0 (0.00%)
Missing	0 (0.00%)	1611 (100.00%)
Circumcised at baseline		
No	153 (69.86%)	548 (34.02%)
Yes	66 (30.14%)	858 (53.26%)
Missing	0 (0.00%)	205 (12.73%)
Anal sex^a^		
No	211 (96.35%)	1354 (84.05%)
Yes	8 (3.65%)	249 (15.46%)
Missing	0 (0.00%)	8 (0.50%)
Exchange of sex for money/gifts^a^		
No	207 (94.52%)	1350 (83.80%)
Yes	12 (5.48%)	255 (15.83%)
Missing	0 (0.00%)	6 (0.37%)
Sex with alcohol/drug use^a^		
No	138 (63.01%)	648 (40.22%)
Yes	81 (36.99%)	958 (59.47%)
Missing	0 (0.00%)	5 (0.31%)
Number of sex partners^a^		
≤ 1	105 (47.95%)	496 (30.79%)
≥ 2	114 (52.05%)	1115 (69.21%)
Married/has main partner^b^		
No	70 (31.96%)	180 (11.17%)
Yes	149 (68.04%)	1375 (85.35%)
Missing	0 (0.00%)	56 (3.48%)
Lives with spouse/main partner^b^		
No	74 (33.79%)	1096 (68.03%)
Not applicable	70 (31.96%)	180 (11.17%)
Yes	75 (34.25%)	278 (17.26%)
Missing	0 (0.00%)	57 (3.54%)
Sex with HIV + partner^a^		
No	216 (98.63%)	740 (45.93%)
Yes	3 (1.37%)	868 (53.88%)
Missing	0 (0.00%)	3 (0.19%)
Unprotected sex with HIV + partner^a^		
No	219 (100.00%)	761 (47.24%)
Yes/don’t know	0 (0.00%)	22 (1.37%)
Not asked	0 (0.00%)	824 (51.15%)
Missing	0 (0.00%)	4 (0.25%)
Genital discharge		
No	213 (97.26%)	1585 (98.39%)
Yes	6 (2.74%)	22 (1.37%)
Missing	0 (0.00%)	4 (0.25%)
Genital sores		
No	215 (98.17%)	1583 (98.26%)
Yes	4 (1.83%)	26 (1.61%)
Missing	0 (0.00%)	2 (0.12%)
Positive for Syphilis, *Neisseria gonorrhoeae*, or *Chlamydia trachomatis*; HVTN 702 only		
No	0 (0.00%)	1016 (63.07%)
Yes	0 (0.00%)	228 (14.15%)
Missing	219 (100.00%)	367 (22.78%)
Sexually transmitted infection^c^ ; HVTN 702 only		
Syphilis	0 (0.00%)	24 (1.93%)
* Neisseria gonorrhoeae*	0 (0.00%)	38 (3.05%)
* Chlamydia trachomatis*	0 (0.00%)	199 (15.98%)
Missing	219 (100.00%)	366 (22.72%)
HSV2 infection; HVTN 503 only		
No	186 (84.93%)	0 (0.00%)
Yes	32 (14.61%)	0 (0.00%)
Missing	1 (0.46%)	1611 (100.00%)

Number (percent) in each category

^a^Timeframe for question is previous 30 days in HVTN 702 and previous 6 months in HVTN 503

^b^In HVTN 702, question was introduced after study began and asked retrospectively when required; 48 MITT males were lost to follow-up prior to its introduction in the study

^c^Denominator is number of participants tested

In HVTN 503, including the extended HVTN 503 S follow-up, over a median follow-up period of 6 years, 14 males acquired HIV. In HVTN 702, the median follow-up was 1.78 years before study unblinding on February 19, 2020, and 37 HIV infections accrued. Annual HIV incidence among placebo-recipient males in HVTN 503 was 1.39% (95% CI 0.76, 2.32). Slightly lower annual HIV incidence was seen ~ 10 years later among males in HVTN 702: 1.33% annually in placebo-recipient males (95% CI 0.80, 2.07) and 1.25% in vaccine-recipient males (95% CI 0.74, 1.97).

### Several Baseline Clinical and Behavioral Factors were Univariately Associated with HIV Risk

When assessed univariately and after accounting for multiplicity, only three baseline variables were found to predict risk of HIV in the pooled HVTN 503 and HVTN 702 data (Tables 3, 4). The strongest predictor by far was not identifying as heterosexual, associated with a 16.23-fold increase in HIV acquisition risk (HR 16.23, 95% CI 8.13, 32.41; adjusted p < 0.01). Anal sex was also associated with an estimated 6.32-fold increase in risk (HR 6.32, 95% CI 3.44, 11.62; adjusted p < 0.01). Exchange of sex for money or gifts was associated with an estimated 3.42-fold increase in risk (HR 3.42, 95% CI 1.80, 6.50; adjusted p < 0.01). Age was not significantly associated with risk (HR 1.33 for age 18–21 vs. 22–25 years, adjusted p = 1; HR 1.22 for 26–35 years vs. 22–25 years, adjusted p = 1).

**Table 3:** Univariate Cox Regression results for baseline demographic variables, stratified by study and treatment arm

Category	HR (95% CI)	Adjusted p-value
Age, years		
18–21	1.33 (0.62–2.85)	1
22–25	–	
26–35	1.22 (0.60–2.50)	1
Race^a^		
Black-only	–	
Not Black-only	3.26 (0.76-14.00)	1
Body mass index (BMI)		
< 18.5	1.58 (0.48–5.25)	1
18.5 to <25	1.23 (0.42–3.59)	1
25 to <30	–	
≥ 30	2.41 (0.52–11.24)	1
Study site geographic region		
Central	2.38 (1.01–5.59)	1
KZN	–	
West/East Cape	2.27 (0.82–6.30)	1

^a^The category ‘Black-only’ consists of participants who identified themselves as single race and Black. ‘Not Black-only’ consists of those who either did not identify as Black, or identified as Black and one or more other races

**Table 4:** Univariate Cox Regression results for baseline behavioral and clinical variables, stratified by study and treatment arm

Category	HR (95% CI)	Adjusted p-value
Heterosexual sexual orientation		
Yes	–	
No	16.23 (8.13–32.41)	< 0.01
Circumcised at baseline		
Yes	–	
No	1.68 (0.88–3.20)	1
Anal sex^a^		
No	–	
Yes	6.32 (3.44–11.62)	< 0.01
Exchange of sex for money/gifts^a^		
No	–	
Yes	3.42 (1.80–6.50)	< 0.01
Sex with alcohol/drug use^a^		
No	–	
Yes	1.14 (0.64–2.04)	1
Number of sex partners^a^		
≤ 1	–	
≥ 2	2.52 (1.23–5.15)	0.07
Married/has main partner^b^		
Yes	–	
No	1.85 (0.91–3.75)	1
Lives with spouse/main partner^b^		
Yes	–	
No	1.07 (0.49–2.30)	1
Not applicable	N/A	1
Sex with HIV + partner^a^		
No	–	
Yes/don’t know	1.60 (0.82–3.16)	1
Unprotected sex with HIV + partner^a^		
No	–	
Not asked	1.80 (0.90–3.60)	1
Yes/don’t know	0.00 (0.00–Inf)	1
Genital sores		
No	–	
Yes	2.04 (0.48–8.74)	1
Genital discharge		
Yes	–	
No	1.03 (0.13–7.89)	1
Positive for Syphilis, *Neisseria gonorrhoeae*, or *Chlamydia trachomatis*^c^		
No	–	
Yes	2.76 (1.13–6.71)	0.36
HSV2 infection^d^		
No	–	
Yes	1.73 (0.42–7.07)	1

^a^Timeframe for question is previous 30 days in HVTN 702 and previous 6 months in HVTN 503

^b^In HVTN 702, question was introduced after study began and asked retrospectively when required; 48 MITT males were lost to follow-up prior to its introduction in the study

^c^Only collected in HVTN 702

^d^Only collected in HVTN 503

*Pre-specified multivariate model identified sexual orientation as the strongest predictor of HIV acquisition. Those not identifying as heterosexual experience higher rates of HIV acquisition*.

Based on a pre-specified multivariate model (Table 5), the only variable that was found to predict HIV risk significantly in the context of the other variables was not identifying as heterosexual, which in the multivariate model had an estimated 14.99-fold increase in risk (HR 14.99, 95% CI 4.99, 45.04; p < 0.01). Having an STI (syphilis, *Neisseria gonorrhoeae*, or *Chlamydia trachomatis*, HR 1.97, 95% CI 0.78, 5.01, p = 0.14), reporting two or more sexual partners (HR 1.95, 95% CI 0.90, 4.22, p = 0.09), exchange of sex for money or gifts (HR 1.67, 95% CI 0.78, 3.55, p = 0.18) and older age (26–35 years vs. 22–25 years, HR 1.87, 95% CI 0.89, 3.93, p = 0.10) had borderline significant associations with HIV. Younger age was a non-significant predictor of risk (HR 1.17 for age 18–21 vs. 22–25 years, p = 0.70). Effect sizes were similar when observed data were used, and participants’ missing data were omitted from the analysis (see Supplementary Materials).

**Table 5:** Multivariate Cox Regression results for baseline variables, stratified by study and treatment arm

Category	HR (95% CI)	p-value
Age, years		
18–21	1.17 (0.52–2.62)	0.70
22–25	–	
26–35	1.87 (0.89–3.93)	0.10
Number of sex partners^a^		
≤ 1	–	
≥ 2	1.95 (0.90–4.22)	0.09
Exchange of sex for money/gifts^a^		
No	–	
Yes	1.67 (0.78–3.55)	0.18
Anal sex^a^		
No	–	
Yes	0.86 (0.29–2.52)	0.77
Sex with alcohol/drug use^a^		
No	–	
Yes	0.86 (0.46–1.59)	0.62
Circumcised at baseline		
Yes	–	
No	1.20 (0.61–2.36)	0.58
Heterosexual sexual orientation		
Yes	–	
No	14.99 (4.99–45.04)	< 0.01
Positive for Syphilis, *Neisseria gonorrhoeae*, or *Chlamydia trachomatis*^b^		
No	–	
Yes	1.97 (0.78–5.01)	0.14

^a^Timeframe for question is previous 30 days in HVTN 702 and previous 6 months in HVTN 503

^b^Only collected in HVTN 702

**Fig. 1:**
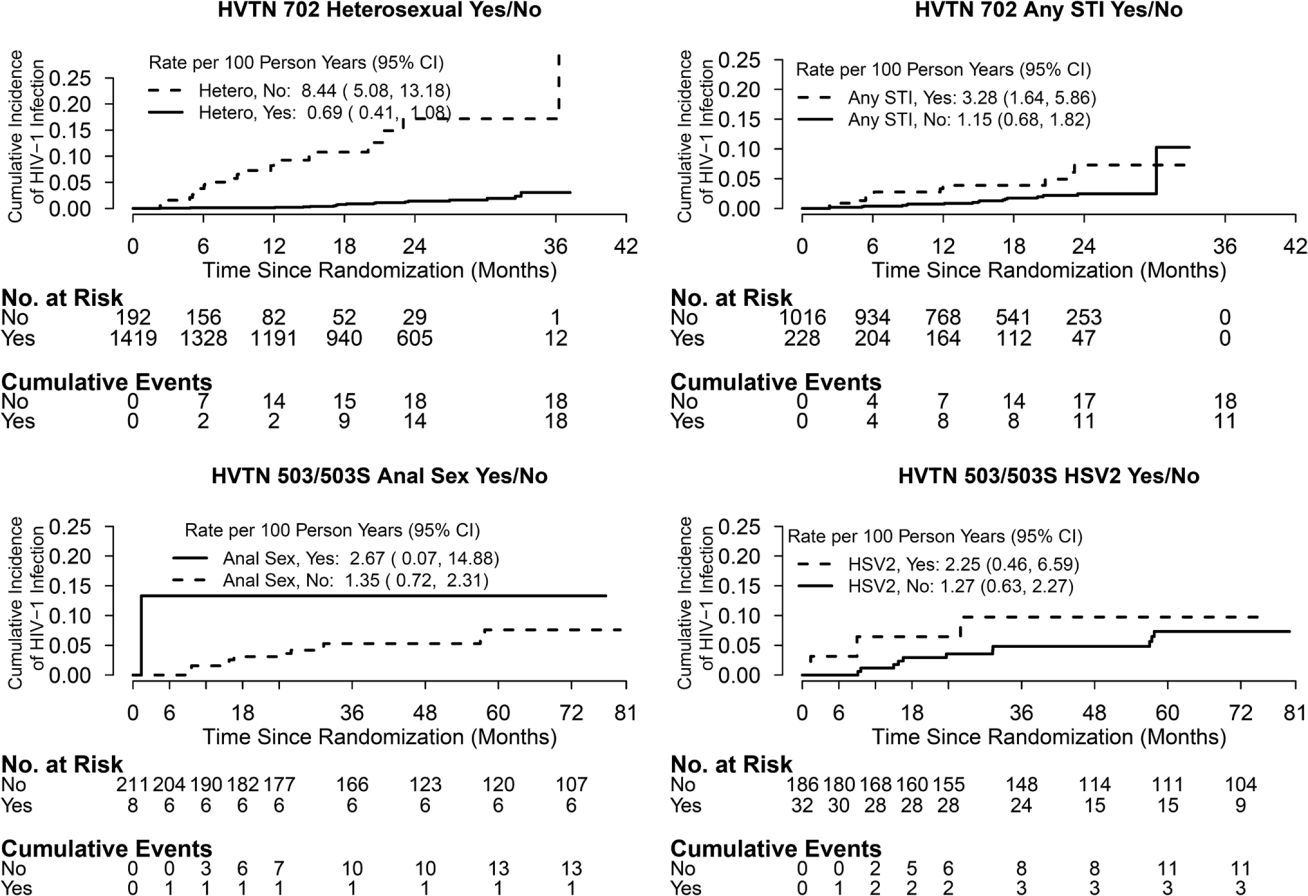
Cumulative incidence of HIV infection by sexual orientation and diagnosis of syphilis, *Neisseria gonorrhoeae*, or *Chlamydia trachomatis* at baseline for HVTN 702 and by anal sex and diagnosis of HSV2 at baseline for HVTN 503 in males

HIV incidence among males with these risk factors was exceptionally high (Fig. 1). In the HVTN 503 participants in this analysis, one of the two males reporting having male sexual partners became HIV-infected and one of the eight males reporting anal intercourse became HIV-infected. In HVTN 702, annual HIV incidence was 8.10% among males not identifying as heterosexual (95% CI 4.96, 12.35). In HVTN 503, annual HIV incidence was 2.23% among males who had tested HSV-2 positive at enrollment (95% CI 0.46, 6.38). In HVTN 702, annual HIV incidence was 3.22% among males who had tested positive for syphilis, *Neisseria gonorrhoeae*, or *Chlamydia trachomatis* at enrollment (95% CI 1.62, 5.69).

For comparison, HIV incidence among mITT females was 4.15% in HVTN 702 and 4.92% in HVTN 503/503S female placebo recipients.

### Machine Learning Analyses Did Not Identify Variables or Combinations of Variables that Better Predicted HIV Risk

**Fig. 2:**
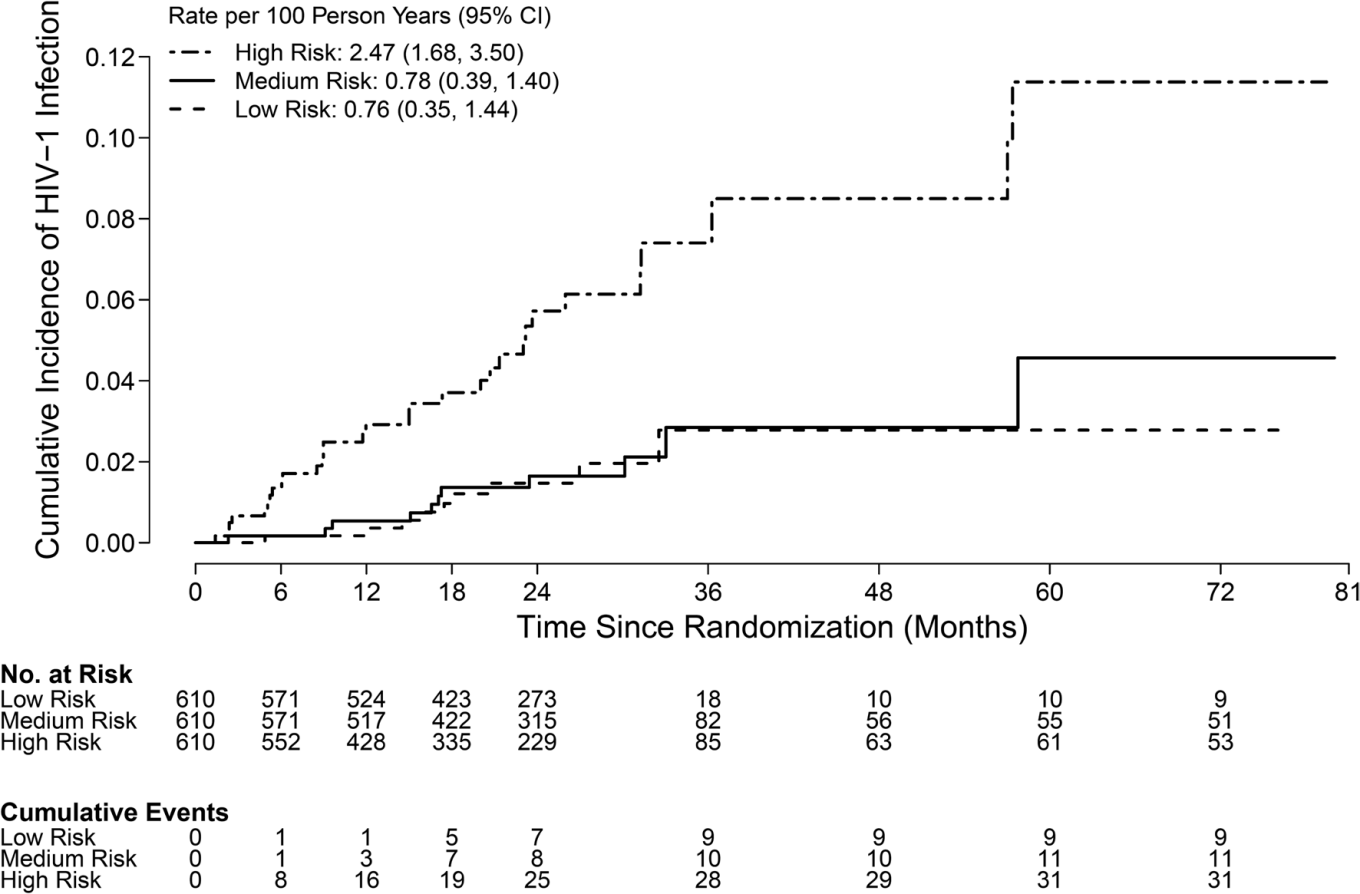
Cumulative incidence of HIV infection in males by tertiles (low, medium, high risk) of the HIV risk score developed using the super-learner method

Super learning identified a risk score algorithm that provided modest ability to predict HIV infection status. The score is a weighted combination of multiple models (‘learners’), each of which involves numerous baseline variables. The two constituent learners with the highest weights are a logistic regression model with LASSO penalty (median weight 0.429 out of 1) and a logistic regression model that first screens variables for univariate associations (median weight 0.282 out of 1). Eight variables were frequently included in this learner across imputed datasets (Supplementary Table S6): exchange of sex for money/gifts, married/has main partner, anal sex, circumcision status, Kwa-Zulu Natal region, sexual orientation, baseline STIs (syphilis, *Neisseria gonorrhoeae*, or *Chlamydia trachomatis*), and number of sexual partners. Six of these variables were included in the pre-specified multivariate model; the latter also includes age and sex with alcohol/drug use. The median cross-validated AUC of the risk score across imputed datasets was 0.70 (95% CI 0.65–0.76), highlighting the variability in performance across imputed datasets. This median performance compares favorably with that of published HIV risk scores for women which have estimated AUCs ranging from 0.56 to 0.73 [17–21]. The risk score successfully identifies a high-risk subgroup with over 8% cumulative HIV incidence over 3 years (Fig. 2).

## Discussion

Our analysis found that, although annual HIV incidence was lower overall amongst males compared to females, specific male subgroups experienced higher annual HIV incidence even when compared to females overall. Males not identifying as heterosexual experienced an almost 15-fold increase in HIV acquisition risk over males identifying as heterosexual. Our finding supports the ongoing prioritization of African men who do not identify as heterosexual for tailored HIV prevention and treatment services. Our findings also support the inclusion of other key male subgroups—men who engage in anal or transactional sex—into prevention efforts in South Africa.

Epidemiological insights into HIV incidence have guided prevention and treatment services toward African women. However, some authors have cautioned that initiatives to prevent and treat HIV amongst males ought not to lag [22, 23]. Amongst multiple clinical, behavioral, and demographic variables analyzed in our study, we found that men who did not identify as heterosexual had the highest rates of HIV acquisition. Our findings are in line with regional studies which found that HIV acquisition in men was associated with several variables, including multiple sexual partners, transactional sex, having an STI, older age, and not identifying as heterosexual [10, 24, 25]. Older age in men, while strongly associated with increased HIV acquisition in other studies, had a borderline association in our study, potentially in part because this cohort enrolled participants over a limited age range (18–35 years; mean age 22 years).

It has been argued that strategically tailoring prevention interventions toward populations which experience high disease burden could optimize benefit and cost-effectiveness [26]. Our data provide behavioral, demographic, and clinical risk factor information to inform such tailored intervention efforts. The differentiated service delivery model, which tailors care according to individuals, has shown promise in improving male engagement in HIV treatment access. The underlying reason seemed to be that it enhanced social “masculine identities” of reputation, respectability and responsibility [27]. It is unclear if non-heterosexual males would value these same masculine identities to promote HIV treatment or prevention service uptake. Indeed, there is a possibility that, in the absence of gender-transformative education, services for males may marginalize those who do not identify as heterosexual [27]. Furthermore, some countries still criminalize non-heterosexual identity, which may limit the reach of differentiated service delivery models.

Older age, a prevalent STI, reporting two or more sexual partners, and exchange of sex for money or gifts had borderline significant associations with HIV in the multivariate model. Machine learning identified a larger set of variables that contributed towards predicting HIV status; predictive performance of the resultant HIV risk score was modest.

We observed similar HIV incidence in the lowest and middle tertiles of risk scores, implying that future phase 1 HIV prevention trials could reasonably exclude only the males most vulnerable to HIV, instead of the current practice of excluding everyone who is vulnerable to HIV [28]. It may be constructive in future efficacy trials to focus enrollment on males at birth exhibiting the aforementioned high-risk characteristics.

This analysis had a few limitations. The results may not be generalizable to all men, because the trials specifically enrolled men deemed to be at risk of HIV acquisition, and we also did not include all enrolled males because of reasons already explained. HVTN 702 and HVTN 503 did not collect some of the data variables identically: the main differences were STIs (HSV2 antibody status was measured in HVTN 503; *Neisseria gonorrhoeae*, *Chlamydia trachomatis* and syphilis testing were conducted in HVTN 702), gender identity, sexual orientation, and timeframe for sexual behavior recall (6 months in HVTN 503 versus 30 days in HVTN 702). To account for these limitations, we made accommodations for missing data. Additionally, although HIV testing was conducted for an additional 10 months in HVTN 702 (46 months versus 36 months in HVTN 503), median follow-up was 1.78 years, compared with 6 years in HVTN 503.

## Conclusion

HIV risk factors for South African males in this cohort are distinct from those described previously for females. For South African males in this analysis, non-heterosexual identity is the most important predictor of HIV acquisition risk. It is therefore appropriate that prevention efforts in South Africa, although focused on the severe epidemic in young women, also encompass key male populations, including men who have sex with men, but also men who engage in anal or transactional sex.

## Supplementary Information

Below is the link to the electronic supplementary material.

Supplementary file1 (PDF 344 kb)
