# Supplementary material for: Risk Factors Associated with HIV Acquisition in Males Participating in HIV Vaccine Efficacy Trials in South Africa
Source: AIDS Behav. 2023 Mar 16;27(9):3027–37. doi: 10.1007/s10461-023-04025-z (PMC10386918; doi:10.1007/s10461-023-04025-z)
Supplement: Supplementary file 1 — Supplementary file1 (PDF 344 kb) [file 10461_2023_4025_MOESM1_ESM.pdf]

Risk factors associated with HIV transmission in men participating in HIV vaccine trials in South Africa: the HVTN 702 Uhambo and HVTN 503 Phambili trials

13 December 2022

Contents

1 CONSORT Diagram **2**

2 Summary of literature supporting multivariate model **3**

3 Details of imputation procedure **3**

4 Supplementary Tables **4**

5 Super-learner methods and supplementary results **7**

1 CONSORT Diagram

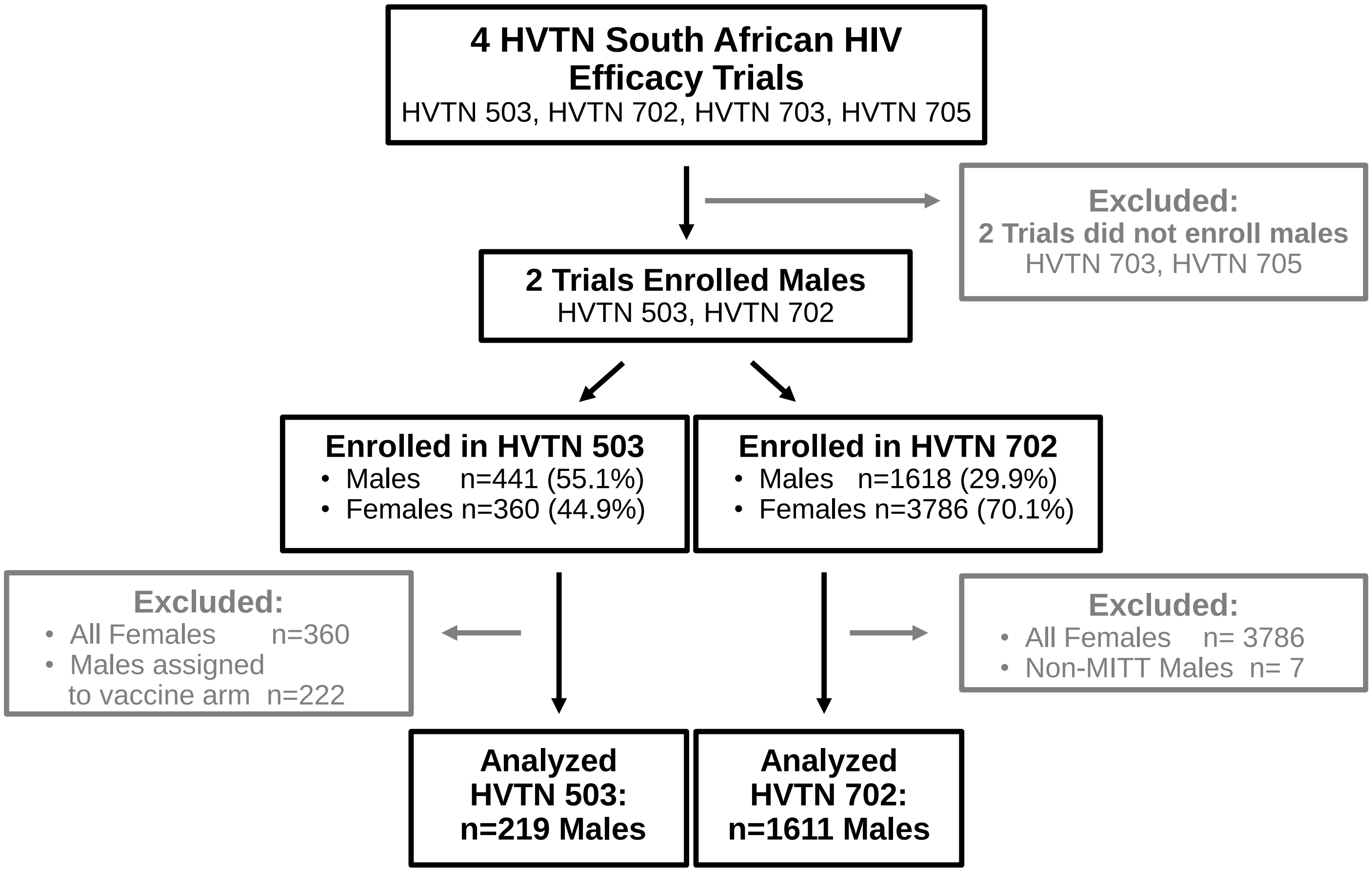

Figure S1: CONSORT diagram for the analysis population

2 Summary of literature supporting multivariate model

A literature review was conducted to assess prior evidence of HIV risk factors in African men. If a risk factor was found to be statistically significant predictor of HIV based on a multivariate model in at least two papers, it was added to the list of published risk factors. Published risk factors that were measured in HVTN 702 and 503 in some form - although not necessarily over the same time period or with the same categories - were included in the pre-specified multivariate model.

The table below lists the variables in the pre-specified multivariate model, and the papers that identified these as predictors of HIV in African men.

Table S1: Literature sources supporting variables included in the multivariate Cox model

Risk.Factor	Source	Variable
Age	Baral et al. BMC Public Health 2011<br>Giorgio et al. AIDS Behav. 2017<br>Sandfort et al. AIDS Behav. 2015<br>Govender et al. BMJ Open 2019<br>Jewkes et al. Int. Jrnl of Epi 2006<br>Lane et al. AIDS Behav. 2011	Age
Number of sexual partners	Baral et al. BMC Public Health 2011<br>Sandfort et al. AIDS Behav. 2015<br>Lane et al. AIDS Behav. 2011	Number of sexual partners
Transactional sex	Baral et al. BMC Public Health 2011<br>Giorgio et al. AIDS Behav. 2017<br>Sandfort et al. AIDS Behav. 2015<br>Lane et al. AIDS Behav. 2011	Exchange of sex for money/gifts
Unprotected anal sex	Lane et al. AIDS Behav. 2011<br>Rispel et al. J Acquir Immune Defic Syndr. 2011	Anal sex
Alcohol use/ hazardous drinking	Rehm et al. Addiction 2016<br>Giorgio et al. AIDS Behav. 2017	Sex with alcohol/drug use
Not circumcised	Govender et al. BMJ Open 2019<br>Jewkes et al. Int. Jrnl of Epi 2006	Not circumcised
Sex with man or not identifying as heterosexual	Lane et al. AIDS Behav. 2011<br>Jewkes et al. Int. Jrnl of Epi 2006<br>Burrell et al. Sexual Health 2010<br>Rispel et al. J Acquir Immune Defic Syndr. 2011	Not identifying as heterosexual
Prevalent STI by self-report/diagnosis	Burrell et al. Sexual Health 2010<br>Rispel et al. J Acquir Immune Defic Syndr. 2011	Prevalent STI diagnosis

3 Details of imputation procedure

Missing baseline variables were imputed using the R package ‘mice’. The package imputes categorical variables using polytomous regression, binary variables using logistic regression, and continuous variables using predictive mean matching. The entire set of baseline variables (Tables 1 and 2) and HIV outcomes was used as the basis for the imputation. A total of 100 imputed datasets were generated and results were combined across imputed datasets using Rubin’s rules.

4 Supplementary Tables

Table S2: Distribution of baseline variables among MITT males by heterosexual (collected in 702)/no male partner reported (collected in 503/503S) or not heterosexual/male partner(s) reported

Category	Not Heterosexual/Male Partner(s)	Heterosexual/No Male Partner
MITT males	194	1636
Age, years		
18-21	74 (38.14%)	344 (21.03%)
22-25	72 (37.11%)	429 (26.22%)
26-35	48 (24.74%)	863 (52.75%)
Median (25%ile, 75%ile)	22 (20, 25)	26 (22, 30)
Race		
Asian	0 (0.00%)	1 (0.06%)
Black	189 (97.42%)	1613 (98.59%)
Colored/Mixed	4 (2.06%)	14 (0.86%)
Multiple reported	0 (0.00%)	4 (0.24%)
Other	1 (0.52%)	2 (0.12%)
White	0 (0.00%)	2 (0.12%)
Body mass index (BMI)		
<18.5	28 (14.43%)	250 (15.28%)
18.5-<25	127 (65.46%)	1147 (70.11%)
25-<30	24 (12.37%)	184 (11.25%)
>=30	15 (7.73%)	55 (3.36%)
Region Categorization of Enrollment Site		
Central	153 (78.87%)	851 (52.02%)
KZN	12 (6.19%)	525 (32.09%)
West/East Cape	29 (14.95%)	260 (15.89%)
Circumcised at baseline		
Yes	96 (49.48%)	828 (50.61%)
No	90 (46.39%)	611 (37.35%)
Missing	8 (4.12%)	197 (12.04%)
Anal Sex*		
No	19 (9.79%)	1546 (94.50%)
Yes	175 (90.21%)	82 (5.01%)
Missing	0 (0.00%)	8 (0.49%)
Exchange of sex for money/gifts*		
No	112 (57.73%)	1445 (88.33%)
Yes	81 (41.75%)	186 (11.37%)
Missing	1 (0.52%)	5 (0.31%)
Sex with Alcohol/Drug Use*		
No	63 (32.47%)	723 (44.19%)
Yes	131 (67.53%)	908 (55.50%)
Missing	0 (0.00%)	5 (0.31%)
Number of sex partners*		
<=1	33 (17.01%)	568 (34.72%)
>=2	161 (82.99%)	1068 (65.28%)
Married / has main partner+		
Yes	141 (72.68%)	1383 (84.54%)
No	52 (26.80%)	198 (12.10%)
Missing	1 (0.52%)	55 (3.36%)
Lives with spouse/main partner+		
Yes	22 (11.34%)	331 (20.23%)

Table S2: Distribution of baseline variables among MITT males by heterosexual (collected in 702)/no male partner reported (collected in 503/503S) or not heterosexual/male partner(s) reported *(continued)*

Category	Not Heterosexual/Male Partner(s)	Heterosexual/No Male Partner
No	119 (61.34%)	1051 (64.24%)
Not Applicable	52 (26.80%)	198 (12.10%)
Missing	1 (0.52%)	56 (3.42%)
Sex with HIV+ Partner*		
No	55 (28.35%)	901 (55.07%)
Yes	139 (71.65%)	732 (44.74%)
Missing	0 (0.00%)	3 (0.18%)
Unprotected Sex with HIV+ Partner*		
No	58 (29.90%)	922 (56.36%)
Yes/Don't Know	3 (1.55%)	19 (1.16%)
Not Asked	132 (68.04%)	692 (42.30%)
Missing	1 (0.52%)	3 (0.18%)
Genital Sores		
No	192 (98.97%)	1606 (98.17%)
Yes	2 (1.03%)	28 (1.71%)
Missing	0 (0.00%)	2 (0.12%)
Genital Discharge		
No	189 (97.42%)	1609 (98.35%)
Yes	4 (2.06%)	24 (1.47%)
Missing	1 (0.52%)	3 (0.18%)

* Timeframe for question is previous 30 days in HVTN 702 and previous 6 months in HVTN 503.

+ In HVTN 702, question was introduced after study began and asked retrospectively when required; 48 MITT males were lost to follow-up prior to its introduction in the study.

^ Denominator used for STIs is number of people tested.

[1] $ Positive for one or more sexually transmitted infection among: Syphilis, Neisseria gonorrhoeae, Chlamydia trachomatis or HSV2

Table S3: Multivariate analysis to characterize association of baseline variables with HIV risk stratified by study and treatment arm. Model fit to complete observed data. All HVTN 702 and HVTN 503/503S follow-up data are included.

Category	HR (95% CI)	p-value
Age, years		
18-21	1.58 (0.66- 3.78)	0.30
22-25	-	-
26-35	1.68 (0.72- 3.90)	0.23
Number of sex partners		
<=1	-	-
>=2	1.88 (0.81- 4.34)	0.14
Exchange of sex for money/gifts		
No	-	-
Yes	1.90 (0.86- 4.20)	0.11
Anal sex		
No	-	-
Yes	1.42 (0.43- 4.70)	0.57
Sex with alcohol/drug use		
No	-	-
Yes	1.09 (0.54- 2.20)	0.80
Circumcised at baseline		
Yes	-	-
No	1.58 (0.79- 3.16)	0.20
Heterosexual/no male partner		
Yes	-	-
No	11.02 (2.96-40.99)	<0.01
Positive for one or more STIs$		
No	-	-
Yes	2.43 (1.20- 4.93)	<0.05

$ Positive for one or more sexually transmitted infections among: Syphilis, Neisseria gonorrhoeae, Chlamydia trachomatis and HSV2

5 Super-learner methods and supplementary results

The same variables considered in the regression models we considered as covariates in the nonparametric ensemble-based cross-validated learning, also known as Super-learning, and used to build an HIV risk score. The risk score is defined as the logit of the predicted HIV infection probability from a regression model estimated using the ensemble algorithm Superlearner, where this logit predicted outcome is scaled to have empirical mean zero and empirical standard deviation one.

Super-learning was implemented on each of the 100 imputed datasets. Seven different learners were included in the learner library: a mean model (no predictors), logistic regression, logistic regression with all two-way interactions between variables, logistic regression with lasso penalty implemented using glmnet, logistic generalized additive model implemented using gam, boosted logistic regression implemented using xgboost, and random forest implemented using ranger. All of the selected learners are coded into the SuperLearner R package available on CRAN. The learners all model the HIV outcome as binary and treat censored subjects as HIV-uninfected; this simplification is reasonable given the low HIV incidence, and binary outcome and censored data methods have been seen to produce similar results in other analyses of these data.

The learners were implemented with different approaches to variable pre-screening: all variables eligible for inclusion, including variables with non-zero coefficients in a lasso fit, including variables with univariate Wald test 2-sided p-values in logistic regression < 0.10, and selecting only one variable at random from amongst a pair of quantitative variables with pairwise Spearman rank correlation > 0.90. Supplementary Table S4 lists the learner-screen combinations that were considered (14 in total).

For each of the 100 imputed datasets, Superlearner was implemented after pre-scaling each quantitative and ordinal variable to have mean 0 and standard deviation 1. Two levels of cross-validation were used: 1) Outer level: a cross-validated AUC (CV-AUC) was computed over 5-fold cross-validation, and 2) Inner level: 5-fold CV was used to estimate weights associated with each learner in the ensemble. Results were summarized across the 100 imputed datasets using mean, median, and standard deviation.

The weights associated with each constituent learner are reported in Supplementary Table S5. The coefficients of each of the variables in each constituent learner are in Supplementary Table S6.

Classification accuracy of different models was measured using CV-AUC (Hubbard et al., 2016; Williamson et al., 2020) as estimated using the R package vimp available on CRAN. CV-AUC values for constituent learners and the Super-learner model are in Supplementary Table S7 .

To estimate the predictive ability of Superlearner on out-of-sample test data, Super-learning was also implemented on each of the 100 imputed datasets by splitting them randomly into 2:1 train:test sets. Each split was stratified by HIV infection status to ensure 2:1 representation of HIV cases in all train:test sets. The Superlearner model and all constituent learners developed using the training set were subsequently used to predict outcome probability on the test set, and AUC was used to measure performance. Median AUC of the risk score across imputed test sets was 0.688 [95% CI: 0.555 - 0.778], which was comparable to CV-AUC. This suggested that the CV-AUC is a good estimate of out-of-sample performance.

References

Hubbard, A.E., Khered-Pajouh, S. and van der Laan, M.J. (2016), “Statistical inference for data adaptive target parameters”, The International Journal of Biostatistics, 12, 3-19.

Williamson, B.D., Gilbert, P.B., Simon, N.R. and Carone, M. (2020), “A unified approach for inference on algorithm-agnostic variable importance”, arXiv preprint arXiv:2004.03683.

Table S4: All learner-screen combinations (14 in total) used as input to the Superlearner.

**Learner**	**Screen***
SL.mean	all
SL.glm	all<br>glmnet<br>univar_logistic_pval<br>highcor_random
SL.glm.interaction	glmnet<br>univar_logistic_pval<br>highcor_random
SL.glmnet	all
SL.gam	glmnet<br>univar_logistic_pval<br>highcor_random
SL.xgboost	all
SL.ranger.imp	all

Note:
*Screen details:
all: includes all variables
glmnet: includes variables with non-zero coefficients in the standard implementation of SL.glmnet that optimizes the lasso tuning parameter via cross-validation
univar_logistic_pval: Wald test 2-sided p-value in a logistic regression model < 0.10
highcor_random: if pairs of quantitative variables with Spearman rank correlation > 0.90, select one of the variables at random

Table S5: Summary statistics (N, mean, median, standard error, and 95% CI) of weights illustrating which constituent learners of non-zero weights assigned by Superlearner to individual learner-screen combinations when run on full dataset and using HIV-1 status as outcome. The N shows number of imputed datasets (out of 100) that a learner is assigned non-zero weight by the Superlearner. Confidence intervals based on 2.5 and 97.5 quantiles from the weights from the 100 datasets.

			Weights			
Learner	Screen	N	Mean	Median	Std. Error	CI
SL.glmnet	all	96	0.408	0.429	0.020	[0.04, 0.706]
SL.glm	univariate_logistic_pval	95	0.326	0.282	0.020	[0.04, 0.76]
SL.xgboost	all	62	0.155	0.127	0.013	[0.006, 0.431]
SL.mean	all	45	0.070	0.066	0.004	[0.01, 0.141]
SL.gam	univariate_logistic_pval	41	0.167	0.155	0.009	[0.009, 0.315]
SL.glm.interaction	highcor_random	39	0.022	0.020	0.001	[0.002, 0.058]
SL.glm.interaction	univariate_logistic_pval	30	0.042	0.036	0.003	[0.003, 0.114]
SL.glm.interaction	glmnet	24	0.126	0.098	0.013	[0.006, 0.468]
SL.glm	all	23	0.143	0.109	0.011	[0.012, 0.342]
SL.glm	glmnet	7	0.133	0.104	0.006	[0.068, 0.22]
SL.gam	glmnet	6	0.121	0.096	0.006	[0.068, 0.217]
SL.ranger.imp	all	4	0.048	0.044	0.003	[0.019, 0.083]

Table S6: Summary statistics (N, mean, median, standard error, and 95% CI) of the odds ratio of predictors in learners assigned weight > 0.0 by Superlearner in any of the 100 imputed datasets. Randomforest and xgboost results reported separately. N learner indicates number of datasets for which the weight was non-zero for the particular constituent learner. N predictor indicates number of datasets for which the weight was non-zero and the predictor was also given a non-zero estimate. Confidence i ntervals b ased o n 2 .5 a nd 9 7.5 quantiles.

				N		Odds Ratio			
**Learner**	**Screen**	**Predictors**	**Max Weight**	**learner**	**predictor**	**Mean**	**Median**	**Std. Error**	**CI**
SL.glmnet	all	hetero	0.828	96	96	0.29	0.251	0.016	[0.157, 1]
SL.glmnet	all	anysti	0.828	96	73	1.487	1.377	0.047	[1, 2.447]
SL.glmnet	all	circ	0.828	96	60	0.886	0.929	0.014	[0.541, 1]
SL.glmnet	all	malepart	0.828	96	28	1.202	1.000	0.061	[0.965, 3.205]
SL.glmnet	all	hsv2	0.828	96	22	1.3	1.000	0.087	[1, 3.519]
SL.glmnet	all	npartm	0.828	96	12	1.044	1.000	0.016	[1, 1.457]
SL.glmnet	all	mainprt	0.828	96	12	0.977	1.000	0.007	[0.759, 1]
SL.glmnet	all	gensor	0.828	96	11	1.084	1.000	0.032	[1, 2.069]
SL.glmnet	all	regcatKZN	0.828	96	11	0.985	1.000	0.005	[0.791, 1]
SL.glmnet	all	raceBlack	0.828	96	11	0.978	1.000	0.008	[0.776, 1]
SL.glmnet	all	exchsx	0.828	96	9	1.019	1.000	0.008	[1, 1.278]
SL.glmnet	all	sxhivp	0.828	96	9	0.97	1.000	0.011	[0.546, 1]
SL.glmnet	all	bmicatgte30	0.828	96	3	0.997	1.000	0.002	[0.973, 1]
SL.glmnet	all	analsx	0.828	96	3	0.99	1.000	0.007	[1, 1]
SL.glmnet	all	regcatWest__EastCape	0.828	96	1	1	1.000	–	–
SL.glmnet	all	bmicat25__lt30	0.828	96	1	0.999	1.000	–	–
SL.glm	univariate__logistic__pval	anysti	0.807	95	95	2.193	2.135	0.067	[1, 3.51]
SL.glm	univariate__logistic__pval	npartm	0.807	95	95	1.573	1.569	0.02	[1, 1.914]
SL.glm	univariate__logistic__pval	exchsx	0.807	95	95	1.205	1.196	0.011	[1, 1.455]
SL.glm	univariate__logistic__pval	livwprtNotApplicable	0.807	95	95	0.833	1.000	0.041	[0.125, 1]
SL.glm	univariate__logistic__pval	mainprt	0.807	95	95	0.758	0.760	0.012	[0.57, 1]
SL.glm	univariate__logistic__pval	regcatKZN	0.807	95	95	0.588	0.569	0.01	[0.5, 1]
SL.glm	univariate__logistic__pval	analsx	0.807	95	95	0.482	0.452	0.017	[0.286, 1]
SL.glm	univariate__logistic__pval	hetero	0.807	95	95	0.201	0.150	0.02	[0.041, 1]
SL.glm	univariate__logistic__pval	circ	0.807	95	92	0.577	0.549	0.015	[0.398, 1]
SL.glm	univariate__logistic__pval	malepart	0.807	95	78	1.51	1.098	0.104	[0.596, 4.382]
SL.glm	univariate__logistic__pval	hsv2	0.807	95	77	1.805	1.397	0.139	[0.331, 4.736]
SL.glm	all	gensor	0.380	23	23	1.495	1.000	0.096	[1, 3.781]
SL.glm	all	npartm	0.380	23	23	1.211	1.000	0.041	[1, 2.203]
SL.glm	all	anysti	0.380	23	23	1.202	1.000	0.045	[1, 2.312]
SL.glm	all	exchsx	0.380	23	23	1.106	1.000	0.023	[1, 1.777]
SL.glm	all	regcatWest__EastCape	0.380	23	23	1.026	1.000	0.006	[1, 1.183]
SL.glm	all	agecatf226__35	0.380	23	23	1.007	1.000	0.005	[0.886, 1.142]
SL.glm	all	livwprtNotApplicable	0.380	23	23	1	1.000	0	[1, 1]
SL.glm	all	bmicatgte30	0.380	23	23	0.955	1.000	0.015	[0.497, 1.055]
SL.glm	all	mainprt	0.380	23	23	0.942	1.000	0.012	[0.627, 1]
SL.glm	all	regcatKZN	0.380	23	23	0.926	1.000	0.014	[0.629, 1]

Table S6: Summary statistics (N, mean, median, standard error, and 95% CI) of the odds ratio of predictors in learners assigned weight > 0.0 by Superlearner in any of the 100 imputed datasets. Randomforest and xgboost results reported separately. N learner indicates number of datasets for which the weight was non-zero for the particular constituent learner. N predictor indicates number of datasets for which the weight was non-zero and the predictor was also given a non-zero estimate. Confidence i ntervals b ased o n 2 .5 a nd 9 7.5 q u antiles. *(continued)*

**Learner**	**Screen**	**Predictors**	**Max Weight**	**learner**	**predictor**	**Mean**	**Median**	**Std. Error**	**CI**
SL.glm	all	bmicat25_lt30	0.380	23	23	0.921	1.000	0.015	[0.58, 1]
SL.glm	all	circ	0.380	23	23	0.89	1.000	0.021	[0.41, 1]
SL.glm	all	raceBlack	0.380	23	23	0.888	1.000	0.021	[0.441, 1]
SL.glm	all	analsx	0.380	23	23	0.874	1.000	0.025	[0.292, 1]
SL.glm	all	sxhivp	0.380	23	23	0.845	1.000	0.031	[0, 1]
SL.glm	all	hetero	0.380	23	23	0.801	1.000	0.037	[0.061, 1]
SL.glm	all	hsv2	0.380	23	22	1.407	1.000	0.125	[0.914, 6.112]
SL.glm	all	malepart	0.380	23	21	1.062	1.000	0.037	[0.459, 2.237]
SL.glm	all	agecatf222_25	0.380	23	10	0.98	1.000	0.006	[0.765, 1]
SL.glm	all	usxhivpNotAsked	0.380	23	9	>1000	1.000	>1000	[1, >1000]
SL.glm	all	livwprtYes	0.380	23	5	1.011	1.000	0.005	[1, 1.193]
SL.glm	all	usxalc	0.380	23	2	0.994	1.000	0.004	[1, 1]

Table S7: Summary statistics (mean, median, standard error, and 95% CI) of CV-AUCs illustrating performance of Superlearner and all learner-screen combinations from the imputed datasets (N=100) for risk score analyses using the full dataset set and HIV-1 status as outcome. Confidence intervals based on 2.5 and 97.5 quantiles from the CV-AUCs from the 100 datasets.

		CV-AUC			
Learner	Screen	Mean	Median	Std. Error	CI
SL.gam	univar_logistic_pval	0.718	0.716	0.002	[0.679, 0.77]
SL.glm	univar_logistic_pval	0.718	0.716	0.002	[0.679, 0.77]
SL	-	0.703	0.702	0.003	[0.649, 0.759]
SL.glm	all	0.702	0.702	0.002	[0.663, 0.75]
SL.gam	highcor_random	0.699	0.698	0.002	[0.661, 0.747]
SL.glm	highcor_random	0.699	0.698	0.002	[0.661, 0.747]
Discrete SL	-	0.697	0.697	0.003	[0.646, 0.751]
SL.glmnet	all	0.696	0.690	0.003	[0.644, 0.751]
SL.xgboost	all	0.696	0.693	0.003	[0.651, 0.752]
SL.gam	glmnet	0.688	0.685	0.003	[0.639, 0.748]
SL.glm	glmnet	0.688	0.685	0.003	[0.639, 0.748]
SL.glm.interaction	glmnet	0.655	0.658	0.003	[0.586, 0.711]
SL.ranger.imp	all	0.631	0.628	0.003	[0.584, 0.692]
SL.glm.interaction	univar_logistic_pval	0.598	0.601	0.004	[0.514, 0.669]
SL.glm.interaction	highcor_random	0.508	0.509	0.003	[0.452, 0.563]
SL.mean	all	0.500	0.500	-	-
